# Replication stress in colorectal cancer stem cells

**DOI:** 10.18632/oncotarget.18045

**Published:** 2017-05-20

**Authors:** Gwenola Manic, Ruggero De Maria, Ilio Vitale

**Affiliations:** Ilio Vitale: Department of Biology, University of Rome “Tor Vergata” and Regina Elena National Cancer Institute, Rome, Italy

**Keywords:** cell cycle checkpoint, DNA replication, mitosis, p53, polyploidy

Mounting evidence indicates that human neoplasms are dynamic, heterogeneous and hierarchical systems maintained by subpopulations of immature, self-renewable and multipotent cells known as cancer stem cells (CSCs). CSCs are considered (1) drivers of tumor initiation, progression and spreading; (2) promoters of chemotherapeutic resistance; and (3) seeds for tumor relapse [1]. Further underscoring the relevance of CSCs in tumor pathology, (cancer) stemness gene signatures have been correlated with poor clinical outcomes [2]. The eradication of CSCs is thus required for an efficient cancer therapy.

Very recently, we performed a study to identify novel anti-CSC compounds to be clinically investigated as standalone agents [3]. For this, we took advantage of a large panel of multicellular spheroids enriched for CSCs that were derived from colorectal cancer (CRC) patient specimens. These tumorspheres, to which we will hereafter refer to as CRC-SCs, were all characterized at genetic and cytogenetic levels [3]. A drug library high-throughput screening on three selected CRC-SCs led to the identification of LY2606368 - a novel checkpoint kinase 1 (CHEK1, best known as CHK1) and CHK2 inhibitor also known as prexasertib [4] - as one potent CRC-SC killing agent [3]. When we extended the analysis to the panel of CSCs, we observed a heterogeneous response, with three groups of LY2606368 sensitivity: high sensitive, medium sensitive and low sensitive/resistant CRC-SCs. We were also able to demonstrate that LY2606368 preferentially depleted the CSC fraction exclusively in responsive (*i.e.*, high or medium sensitive) CRC-SCs. CSC eradication by LY2606368 occurred (1) *in vitro*, as shown by the decrease in the percentage of cells positive for the colorectal CSC markers CD44v6 and ephrin B2 (EFNB2) or displaying high level of WNT activity; and (2) *in vivo*, as proven by the diminished tumor growth potential of LY2606368-treated primary and secondary xenografts derived from CRC-SCs [3].

We then elucidated the mechanisms of CSC killing by LY2606368, showing that it involved the inhibition of CHK1 activity followed by a lethal increase in the levels of replication stress (RS). In more detail, CHK1 inhibition by LY2606368 simultaneously impaired the DNA replication process and the intra-S checkpoint resulting in DNA damage accrual, premature mitoses and cell death [3].

Next, we performed a protein network analysis by reverse-phase protein microarrays (RPPA) finding that LY2606368-responding CRC-SCs presented signs of ongoing RS response, including the phosphorylation of ATM serine/threonine kinase (ATM) and replication protein A2 (RPA2, best known as RPA32), coupled to high basal levels of endogenous DNA damage. Immunohistochemistry analyses on sections from CSC-derived xenografts further confirmed DNA damage response (DDR) overactivation and high RS levels at baseline as biomarkers of the response to LY2606368. By combining genetic and cytogenetic profiles with sensitivity data we also found an association between LY2606368 sensitivity and either mutation(s) of the tumor protein p53 (TP53, best known as p53) or increased chromosomal content (*i.e.*, hyperdiploidy). Accordingly, the constitutive depletion of p53 or the pharmacological induction of whole-genome redoubling sensitized previously resistant CRC-SCs to LY2606368 [3].

**Figure 1:**
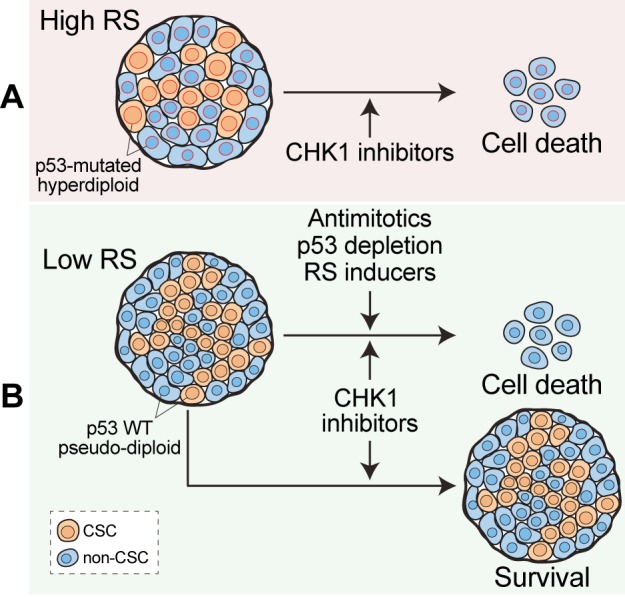
Depleting CRC-SCs by CHK1 inhibition **A.** High levels of replication stress (RS) due to *TP53* mutation (nuclei contoured in red) and/or hyperdiploidy (increased nuclear size) render CRC-SCs targetable with CHK1 inhibitors. **B.** Strategies aimed at boosting RS, increasing ploidy, or abrogating the p53 pathway can sensitize previously resistant CRC-SCs to the inhibition of CHK1.

To sum up, our results indicate that RS in CRC-SCs is promoted by (1) the abrogation of p53 functions, which leads to diminished DNA repair efficiency, unscheduled S-phase entry and/or tolerance to (and protection from cell death induced by) RS; and (2) hyperdiploidy, which increases the risks to incorrectly duplicate the DNA. Of note, high levels of RS make CRC-SCs particularly dependent on the activity of the RS response player CHK1 (Figure 1).

CSCs are reported to share with embryonic and adult SCs a very robust DDR as a means to counter DNA lesions arising from endogenous and exogenous sources [5]. Beyond constituting a barrier to tumorigenesis, the DDR appears indispensable for the survival and fitness of established, genomically-instable tumors thereby representing a candidate anti-cancer target [6, 7]. In our study, we added a further layer of complexity showing that only a subset of CSCs (*i.e.*, those bearing *TP53* mutation(s) and elevated chromosomal content) displayed high basal levels of endogenous DNA damage, which conferred them a peculiar dependency on CHK1 functions. Importantly, our results can pave the way to the entry of CHK1 inhibitors into the clinical practice for the eradication of colorectal tumors presenting replication-stressed, p53-deficient and hyperdiploid CSCs.
